# An Intervention Offering Self-management Support Through mHealth and Health Coaching to Patients With Prostate Cancer: Interpretive Description of Patients’ Experiences and Perspectives

**DOI:** 10.2196/34471

**Published:** 2022-09-08

**Authors:** Louise Faurholt Obro, Palle Jörn Sloth Osther, Jette Ammentorp, Gitte Thybo Pihl, Kasper Kvols Heiselberg, Peter Gall Krogh, Charlotte Handberg

**Affiliations:** 1 Urological Research Center Vejle Hospital (Lillebaelt Hospital) University Hospital of Southern Denmark Vejle Denmark; 2 Department of Regional Health Research Faculty of Health Sciences University of Southern Denmark Odense Denmark; 3 Centre for Research in Patient Communication Odense University Hospital Odense Denmark; 4 Department of Clinical Research University of Southern Denmark Odense Denmark; 5 UCL Vejle Denmark; 6 Department of Engineering Aarhus University Aarhus Denmark; 7 Steno Diabetes Center Aarhus Aarhus University Hospital Aarhus Denmark; 8 National Rehabilitation Center for Neuromuscular Diseases Aarhus Denmark; 9 Department of Public Health Faculty of Health Aarhus University Aarhus Denmark

**Keywords:** mobile phone, mobile health, mHealth, prostate cancer, self-management, health coaching, coaching

## Abstract

**Background:**

Observational management strategies such as active surveillance and watchful waiting are considered to be acceptable approaches in patients with low-risk localized prostate cancer and a safe alternative to aggressive treatment. During observational management, treatment is postponed until the disease progresses, which often never occurs. However, approximately 90% of patients with a low-risk disease choose aggressive treatment owing to anxiety. Strategies to address anxiety are needed for optimal management of this population and to improve the quality of life of patients with low-risk localized prostate cancer. A review highlighted that mobile health (mHealth) in tandem with health coaching can support patients’ self-management of health behaviors and improve well-being.

**Objective:**

This study aims to explore patients’ experiences with and perspectives on an intervention offering self-management support through the use of mHealth devices and health coaching to identify supportive features that enable patients to perform sustainable changes that improve well-being.

**Methods:**

We used an interpretive description approach, combining semistructured interviews with 13 purposively selected patients with prostate cancer and participant observations of patient-coach interactions in coaching sessions. The interviews were transcribed and analyzed. The self-determination theory was used as a theoretical lens. Field notes and coaching notes from each session were used to orient data generation and confirm or challenge the analysis.

**Results:**

Our analysis suggested that patients’ self-awareness and psychological identity influenced their experiences with and perspectives on the self-management support offered by mHealth and health coaching in clinical practice. The patients’ individual experiences and perspectives indicated that they placed themselves in a dynamic continuum of sustaining or repressing their identity, self-awareness, and individual qualities. Our analysis revealed 4 interacting themes, all related to the psychological identity of the patients.

**Conclusions:**

For the group of patients with prostate cancer to experience well-being, we found it important for them to sustain their self-image when offered a self-management intervention. Motivation and autonomy were important aspects for the individual patients to sustain their self-image throughout the intervention. In contrast, demotivation and a sense of paternalism could result in fostering an experience of having to repress self-awareness.

## Introduction

### Background

Prostate cancer (PCa) is the most common malignancy among men [1]. Most men with PCa have low-risk localized PCa, and the cancer is unlikely to become life threatening [2]. Aggressive treatment may provide little survival benefit for patients with low-risk localized PCa while increasing the risk of side effects, such as erectile dysfunction, urinary incontinence, fatigue, weight gain, and depression that may severely impact the quality of life [2]. Observational management strategies such as active surveillance (AS), which is used in a curative setting, and watchful waiting, which is used in a palliative setting, have been shown to be feasible in patients with low-risk localized PCa [3]. Low-risk localized PCa is seen as a chronic disease in which treatment is postponed until the disease progresses, which often never occurs [3]. The observational management strategy involves lifelong plasma prostate-specific antigen testing and, in some cases, repeated biopsies of the prostate [3]. Another important aspect of observational management is to encourage patients to live a healthy and active life because alcohol, smoking, and obesity are among the risk factors for cancer progression [4]. Although observational management appears to be a safe alternative to aggressive treatment, approximately 90% of patients with a low-risk disease choose aggressive treatment [5]. The Prostate Cancer Research International Active Surveillance (PRIAS) study by Bokhorst et al [5] revealed that approximately 13% of patients on AS choose to initiate active treatment for PCa owing to anxiety. Thus, as argued by Bokhorst et al [5], strategies to address anxiety are needed for optimal management and to improve health behaviors and quality of life in patients with low-risk localized PCa. Well-being includes having good mental health, high life satisfaction, a sense of meaning or purpose, and the ability to manage stress and anxiety [6]. Self-management strategies represent a promising approach for treating chronic conditions and improving well-being and quality of life [6].

Mobile health (mHealth) devices, such as smartphones, fitness trackers, and wearables, represent a new generation of tools with the potential to improve patient self-management [6]. mHealth provides reliable and safe data collection outside the clinical setting and facilitates the delivery of interventions (eg, instruction in behavioral change) [6]. Although the promise of mHealth seems appealing, some challenges were highlighted by Woods et al [7], who found that patients often have a significant barrier to using mHealth in everyday life. They found that health care professionals are requested to support patients in adopting technological devices to ease the integration of mHealth as part of self-management strategies [7]. A recent scoping review indicated that mHealth and health coaching work synergistically and enhance patients’ self-management [8].

### Objectives

Health coaching is a patient-centered intervention in which a health coach guides a patient in making behavioral changes. This is typically achieved by encouraging patients’ active participation in self-management based on personal objectives and individual motivational readiness to change [9]. Palmer et al [10] defined health coaching as “a practice of health education and health promotion within a coaching context, to enhance the well-being of individuals, and to facilitate the achievement of their health-related goals.” Accordingly, combining mHealth and health coaching may be a promising approach to support self-management in patients with PCa in observational management. Enabling and empowering patients to assume a more active role in the management of their disease has been shown to increase their quality of life and lower distress [11,12]. Growing evidence suggests that self-management strategies can benefit patients [11,13]. Self-management can be defined as the initiatives undertaken by individuals to promote their health and well-being [14]. It includes the actions individuals take toward a healthy lifestyle: managing their lifelong disease, management of their emotional health and well-being, and prevention of further illness [11,14]. Although several studies point to self-management as a promising strategy for patients to manage their chronic cancer disease [11,14], others address a key issue that needs to be discussed: how health care professionals can support self-management in an evidence-based, structured way and how self-management support can be integrated into clinical practice [15]. Thus, the objective of this study was to explore patients’ experiences with and perspectives on an intervention offering self-management support through different mHealth devices and health coaching to investigate what supported them in making sustainable changes that improved their well-being.

## Methods

### Setting and Sampling

All participants were recruited from the urological outpatient clinic at Vejle Hospital, a part of the Lillebaelt Hospital, Vejle, Denmark. Participants were informed and invited to participate in the study at the urological outpatient clinic by their urologist. We included patients with PCa on AS or watchful waiting who could read and speak Danish. A total of 13 participants were recruited and divided into 2 groups: group 1 between June and August 2017 and group 2 between April and August 2020 (Table 1). The participants were included using purposive sampling [16]. In addition, 4 female urological nurses were included in the study to coach the participants (Table 2). The nurses had completed a 2-day course in health coaching given by a certified health coach.

**Table 1:** Characteristics of the 13 participants including their mobile Health (mHealth) devices and tracked activity.

Group number	Participant number	Recruitment period	Age (years)	Ethnicity	Employment status	Time since diagnosis (years)	mHealth devices	Tracked activity
1	1	June 2017 to August 2017	71	Danish	Retired	5	BTTN^a^	Water intake
1	2	June 2017 to August 2017	70	Danish	Retired	5	BTTN	Water intake
1	3	June 2017 to August 2017	72	Danish	Retired	3	BTTN	Water intake
1	4	June 2017 to August 2017	68	Danish	Self-employed	1	BTTN	Water intake
1	5	June 2017 to August 2017	82	Danish	Retired	6	BTTN	Water intake
1	6	June 2017 to August 2017	70	Danish	Retired	3	BTTN	Water intake
1	7	June 2017 to August 2017	69	Danish	Employed	2	BTTN	Water intake
2	8	April 2020 to August 2020	80	Danish	Retired	2	Fitness tracker and music device	Steps and well-being
2	9	April 2020 to August 2020	81	Danish	Retired	5	Fitness tracker and music device	Steps and well-being
2	10	April 2020 to August 2020	75	Danish	Retired	2	Fitness tracker and music device	Steps and well-being
2	11	April 2020 to August 2020	78	Danish	Retired	3	Fitness tracker and music device	Steps and well-being
2	12	April 2020 to August 2020	72	Danish	Retired	3	Fitness tracker and music device	Steps and well-being
2	13	April 2020 to August 2020	75	Danish	Retired	3	Fitness tracker and music device	Steps and well-being

^a^BTTN: Bluetooth button with connection to the *My Course* app and the electronic patient journal.

**Table 2:** Characteristics of the 4 nurse coaches.

Group number	Nurse number	Recruitment period
1	1	June 2017 to August 2017
1	2	June 2017 to August 2017
2	3	April 2020 to August 2020
2	4	April 2020 to August 2020

### Intervention

**Figure 1:**
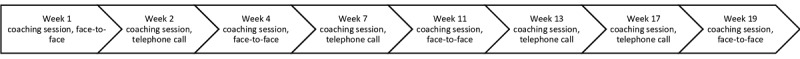
The 19-week coaching program.

All 13 patients participated in a 19-week program that included 8 individual coaching sessions: 4 face-to-face visits to the outpatient clinic, lasting between 45 and 60 minutes each, and 4 telephone calls of 30 minutes each (Figure 1). Participants met the same coach throughout the program. The program aimed to provide ongoing support and guidance for participants to set goals and sustainable objectives and make changes that improved overall health and well-being. In the first coaching session, the participants received an mHealth device that tracked the activity of interest. The tracked activities were related to overall health, well-being, or both. Those related to overall health were physical activity or water intake; they were chosen because they are simple to track and are important aspects of living a healthier life [6]. The tracked activities related to well-being were self-reflection on one’s life, which was chosen because self-reflection is important for good mental health and dealing with emotional challenges [6].

After the first session, all coaching sessions started with the nurse coach and a participant jointly evaluating the data. In addition, difficulties with the mHealth devices were addressed at the beginning of each session. After evaluating the data, the nurse and the participant talked about facilitators and barriers related to the participants’ individual goals and optionally setting new goals. At the end of the session, the nurse summarized the session.

### Group 1

**Figure 2:**
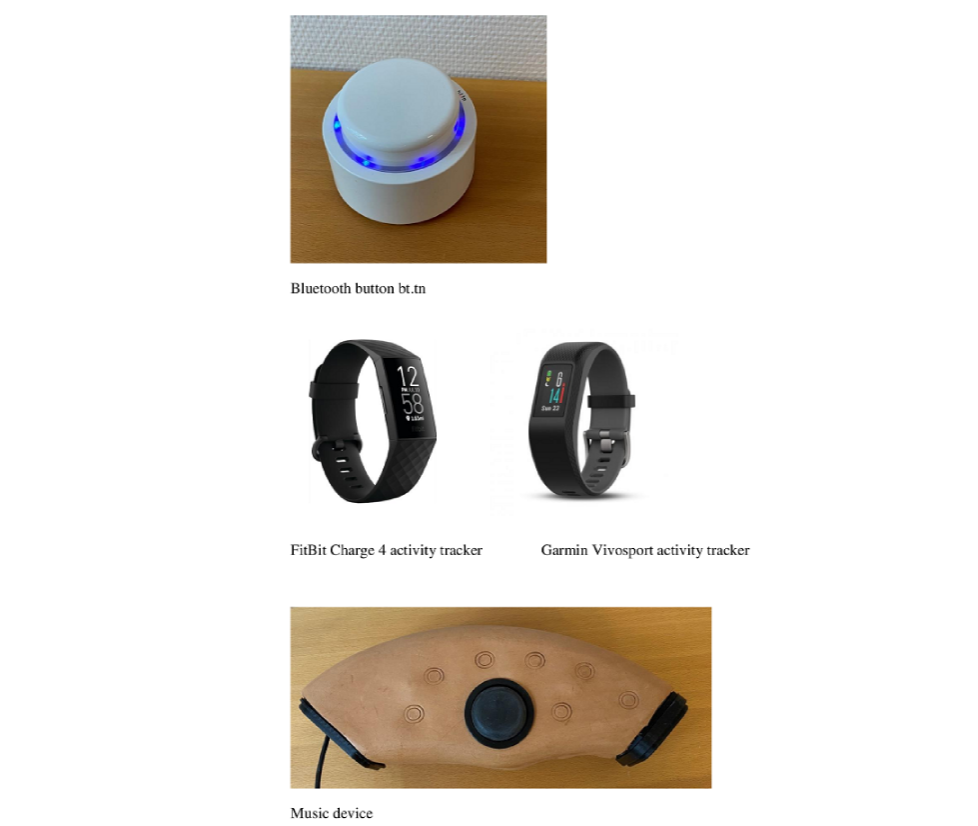
Picture of the mobile health devices.

All participants in group 1 felt that water intake could be improved and agreed to register daily water intake with a Bluetooth button (Figure 2) connected with the My Course app and the electronic patient journal. The participant tracked by a push on the Bluetooth button every time they drank a glass of water. During the following sessions, the coach and the participant evaluated the assessment and discussed the barriers and motivation for achieving the goal of increased water intake.

### Group 2

In the first session, in group 2, the coach and the participants used the *Wheel of Life* [17] to identify and set objectives. The *Wheel of Life* by Meyer [17] is a visual model used in coaching to help clients understand their current sense of balance or fulfillment in life [17]. The wheel, divided into pies, usually consists of 8 to 10 client-identified categories considered important for a full or balanced life [17]. Participants rated their level of satisfaction within each category and then mapped them onto an image of a wheel. This provided them with an immediate overview of their current *life balance* [17]. Subsequently, each category was rated from 1 (not at all satisfied) to 10 (completely satisfied or could not be improved) by drawing a line across the corresponding number in that section of the pie and then shading below it. Completing this leaves the participant and the coach with a jagged wheel that illustrates the areas for growth. Following this exercise, the participants in group 2 were equipped with 2 mHealth devices: a fitness tracker (FitBit or Garmin; Figure 2) and a music device (Figure 2). The participant tracked their steps using a fitness tracker. The objective of the use of a fitness tracker was for the participants to reflect on their daily activities. The objective of the music device was to improve the participants’ well-being by spurring self-reflection. The music device was intended to function as a digital diary in which participants were asked to play a short musical phrase, a fragment of a tune, or something similar that reflected their well-being and emotions on a daily basis. The coach and participants listened to the recordings during the coaching sessions and then discussed what was recorded.

### Methodological Approach

On the basis of the need to explore patients’ experiences with and perspectives on an intervention offering self-management support through mHealth and health coaching and the need to inform and create knowledge in collaboration with clinical practice, we applied the qualitative inductive methodology of interpretive description (ID) [16]. ID was chosen as the methodology because it addresses complex explorative clinical questions while producing practical outcomes [16]. ID applies the notion that social influences are formed by people and from people and their actions; in contrast, it also seeks a nuanced understanding of an individual’s perceptions of the phenomenon of interest [16]. The methodology draws on other qualitative studies such as ethnography, grounded theory, and phenomenology. Furthermore, ID stresses the value of a *research logic*, permitting the researcher to apply and combine the methods needed to fully answer the research question [16]. The flexibility of ID is practical when exploring a field in which unexpected findings may occur, which would require an adjusted strategy [16]. ID allows for early (preliminary) analysis of data that enables mutual adjustment of the data being collected and the models applied for analysis [16]. ID invites a theoretical framework to help structure the study of data material at hand, for example, interview guide, analysis, and discussion [16], and therefore, we also used the self-determination theory (SDT) [18]. We used SDT because it points to how the achievement of health-related goals and well-being is more effective and lasting when patients are autonomously motivated [18]. Patients’ motivation depends on their personal convictions and the degree to which their psychological needs for competence, autonomy, and relatedness are fulfilled [18]. Furthermore, we used SDT to construct the interview guide and conduct interviews and analysis to capture the impact of the mHealth device and health coaching on patients’ psychological needs and motivation.

### Data Collection

Data consisted of semistructured interviews in which a few predefined questions with an open approach were asked, for example, “How did you experience the use of mHealth?” and “What are your perspectives on coaching?” Data also consisted of field notes by the first author (LFO) reporting on observational studies [16] of the coaching sessions and the coaches’ notes from each session, totaling approximately 90 pages. After each coaching session, the coaches wrote notes concerning the patient’s progress, patient’s experiences with the intervention, and possible tasks the patient had opted for. A total of 26 semistructured interviews were conducted by the first author, 13 before the intervention and 13 at the end of the study. The participants were interviewed in the clinic or by phone, and the interview lasted between 30 and 70 minutes. The interviews were audiotaped and transcribed verbatim.

Data from the observations were used to provide insight into coach-participant interactions and to support and elaborate interview data. The observations were made in October 2017 and October 2020 (approximately 5 hours of observations).

### Data Analysis

The analysis was guided by the ID methodology [16]. Interview data were transcribed, anonymized, and transferred to the QRS NVivo software, version 12. In the first step of the analysis, the first author (LFO) and the last author (CH) read the data and developed an initial coding structure based on the initial analysis. LFO and CH then performed a more specific coding strategy, shifting between the process of coding and taking *a* *step back* to gain perspective on the data material as a whole. After the initial coding, LFO and CH further refined, described, and discussed themes grounded in the remaining data. This step was repeated, ensuring that the themes comprised the data, and subsequently, the themes were discussed with the rest of the research team (KH, PGK, JA, and GTP). Correspondingly, we addressed any inconsistencies within and between the interview data, field notes, and notes from the coaches. Finally, we created a model that represented the analytical findings in a hierarchy [16].

### Ethical Considerations

All participants received oral and written information on the purpose and methods of the study, including their right to withdraw at any time. If participants agreed to participate, they signed an informed consent form. All data were anonymized and stored in a secure place that was approved by the Danish Data Protection Agency in accordance with the General Data Protection Regulation. This study was approved by the Regional Committees on Health Research Ethics for Southern Denmark (case ID: 20212000-105).

## Results

### Overview

**Figure 3:**
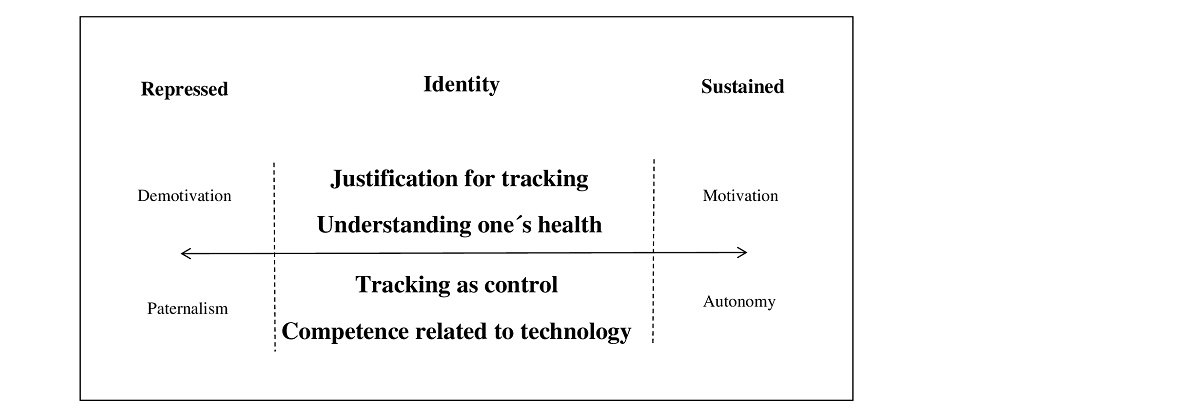
Understanding the perspectives of patients with prostate cancer on an intervention offering self-management support through mobile health and health coaching in clinical practice.

Our analysis revealed that patients’ identity, including their self-awareness and self-image, seemed to influence their experience with and perspective on self-management support through mHealth and health coaching in clinical practice. The patients’ individual experiences and perspectives indicated that they placed themselves in a dynamic continuum of sustaining or repressing their sense of identity. Our analysis revealed four interacting themes related to the identity and self-awareness of the patients (Figure 3): (1) justification for tracking and (2) understanding one’s health collectively shaped a spectrum of patient views ranging from being motivated for self-tracking to being demotivated and (3) tracking as control and (4) competence related to IT ranged in a spectrum from a sense of autonomy to paternalism. An illustration of the findings is presented in Figure 3.

### Justification for Tracking: Motivation and Demotivation

The patients explained their tracking of the intervention by unfolding a spectrum from being motivated to being demotivated. Some patients stated that they were motivated to track the data because they liked the idea of self-tracking. However, some patients explained that they did not find tracking motivating but still tracked data because they liked the interaction with the coach:

I needed to talk to someone about it (the stressful situation at home) and the coach was really nice to talk to, in a sense..., she could see things differently.

P13

The patients who found tracking motivating related it to an opportunity to gain insight into the intervention. One patient explained the following:

The reason I signed up for this was that I got a request, and then I'm probably the kind of person who..., if you can do something to gain insight into how you can manage things better in the future, health care and medical related things, then I’m of the opinion that it is my civic duty to sign up.

P10

During the coaching sessions, some participants enthusiastically demonstrated their devices to the coach and described how some technological aspects could be improved. The coach expressed appreciation for the patients’ contributions, which seemed to motivate them even further.

Some patients described that they were tracking because of a sense of duty, either toward the health professionals or their fellow patients with PCa. The sense of duty was explained by some patients as a motivation, whereas for others, it felt demotivating.

A feeling of demotivation for tracking emerged among patients who were struggling to understand the aim and logic behind the intervention. When they could not comprehend the logic behind the devices, in particular the music device, they expressed feeling demotivated about using the device or only using it to repress a feeling of guilt.

One participant using the music device explained the following:

It gave me nothing. On the contrary, it became a pestilence for me to track every day, because I did not benefit from it. I was just trying to get some little tunes and stuff like that; well, nothing...but the fact that it could provide me with a little feeling of calmness and relaxing, right?

P11

From the quotes and the observations, it appeared that when the participants experienced themselves and their insights as important, they experienced that their self-image was sustained, and they felt motivated.

In contrast, other patients argued that when they could not contribute with valuable insights and felt that they were merely “a bystander” (P9) in the intervention, and thereby were only tracking data because of feelings of guilt and duty, they experienced repression of their self-image and were demotivated.

### Understanding One’s Health: Motivation and Demotivation

Several patients described that participating in the intervention provided them with the ability to better understand their health, which they found motivating. Most of the participants in the group that tracked water intake explained that a balanced water intake was related to their well-being and was a motivational factor for them. The patients in this group expressed that by gaining insight into health through tracking and coaching, they adopted a better rhythm in life. They explained that they became more aware of drinking water throughout the day, instead of drinking the whole amount at the end of the day, entailing frequent toilet visits during the night. One patient explained the following:

I think that I benefitted a lot from participating in the project. It has been great, because I have obtained a stable rhythm, remembering to drink water, which is important when you are my age, right? So, yes it has. It has given me a lot, I would say, not just a little.

P2

It seemed that when the patients had a successful understanding of the importance of water intake, they were often more motivated to set a new health objective for increasing balance and well-being in their lives. A patient explained the following:

After attending this intervention my wife and I became more aware of drinking more water and eating healthier, and this gave us a lot of energy to go for a walk or two.

P7

Some patients also highlighted the importance of interacting with the coach to gain a better understanding of their health. The patient described that the coach helped them translate their tracking data into “a language that I understand” (P6). The coach seemed to play an important role for the patients, turning insights from the data into advice for the patients and keeping them motivated.

The importance of the coach in fostering understanding and motivating the patients can also be seen in the observations. In a coaching session, a patient (P9) seemed depressed and resigned when the coach asked questions about their health and life. During the session, the patient experienced anxiety because of the PCa treatment. He told the coach that he did not trust the urologist to control his illness. The coach wrote the following in her notes: “He (P9) seems nervous for his prostate cancer. Also, occasionally explains thoughts about what it would be like not to live anymore” (C2). The coach also described in her notes that, at every session, she engaged in conversation with him on his history of PCa and treatment. She said that he seemed to be increasingly positive and motivated after each session. During the last interview, the patient explained the following: “The conversations I had with (C2), have given me a positive view on life” (P9). As illustrated in these quotes and observations, some patients were motivated by the conversational support from the coach.

Some patients explained that through the understanding of their health and increased self-awareness gained by participating in the intervention, they were able to sustain their self-image and had more courage to ask questions in the consultations with the urologist in the outpatient clinic. For instance, some patients explained that they were more motivated to ask their urologist questions because they had gained a better understanding of their health. Furthermore, the patients explained that registrations on the mHealth devices could contribute to discussions about nocturia with their urologists. In addition, the patients explained that the tracking provided insights for the urologist into the patients’ everyday lives, which could help individualize their treatment.

However, some patients described the newfound understanding of their health as demotivating. The tracking of their data could, for some, be an unpleasant reminder and awareness of their illness and being ill. Furthermore, it appeared that some participants neglected their disease. In this context, a coach described in her notes that a few patients did not believe that they had been diagnosed with PCa, causing the coach to look up the patients’ journals to confirm the PCa diagnosis. Some patients explained that talking about their illness and health had a negative psychological effect on them. Regarding this, some patients expressed that they did not think that self-tracking and coaching could contribute to any new understanding; conversely, they feared that they would be confused by the tracking data and by talking to the coach.

### Tracking as Control: Autonomy and Paternalism

Some patients reported that they had experienced tracking as a method of control. They described a feeling of control, ranging from autonomy to paternalism. Having control over one’s life and maintaining autonomy are important. The feelings of control and autonomy appeared to be linked to each patient’s previous working life and self-image. Some explained that they held high positions and served important functions in their working lives with high degrees of decision integrity. For these patients, PCa diagnosis led to a loss of control. One patient explained the following:

I’m old policeman and I’ve had have had many employees, and stuff like that. Taken a lot of beatings and so on. This prostate cancer diagnosis fucked me up a little. That was actually the worst part. It got personal.

P3

The loss of control and autonomy for some patients seemed to impact their well-being negatively. However, some patients described self-tracking as a way to regain autonomy and well-being. A patient explained the following:

I really thought I was getting enough exercise during the day, but after I have begun wearing the watch (activity-tracker), I could suddenly see that I did not. Now I can see how many steps I have taken, and then I can judge for myself whether I should go for a long or short walk.

P11

The importance of control and autonomy was also observed during the coaching sessions. In one session, a patient (P8) stated that he had been a lawyer and had always been in control. He emphasized his ability to make decisions for himself regarding data tracking. He appeared relaxed when talking with the coach and explained that he liked the idea of self-tracking and that he saw it as a way for him to control his body.

With the use of mHealth, some patients described that they had gained the ability to make judgments to do something good for themselves. As a result of the intervention, the patients seemed to feel more in control of their behavior and how this control could impact their lives. Furthermore, it appeared that when the use of self-tracking was experienced as meaningful and the patients were tracking out of their own free will without external pressure, they explained a sense of autonomous control, which seemed to increase their well-being.

For some patients, self-tracking could be regarded as an external control, which could lead to a sense of being subjected to paternalism. These patients explained that they experienced self-tracking as a way of being controlled by the coach. In a coaching session, a patient (P12) explained that he could not see how he could benefit from tracking data and that he did not want to wear the fitness tracker. He told the coach that he had his own device at home and that he would use it instead. Likewise, other patients stated that they saw self-tracking as a way for the coach to push them to do something they did not want to do. However, some patients explained that at the beginning of the intervention, they had a sense of being forced to track data but experienced that the tracking became more autonomous at the end of the intervention. A patient said the following:

I did it to satisfy the coach. She told me to try recording small melodies. I felt like I had to do it...but in the end it might have given me something.

P10

It appeared that self-tracking could bring autonomy, control of one’s life, and increase well-being when tracking was experienced as meaningful and without an external paternalistic control.

### Competence Related to Technology: Autonomy and Paternalism

The patients described how competencies related to technology ranged from experiencing autonomy to paternalism. All patients had their own smartphone and other electronic devices such as tablets, computers, or smartwatches. Many patients said that they used their smartphone or other devices for several hours each day. The patients explained that they used their smartphone to text, help with wayfinding, read the newspaper, and watch movies. When patients rated their competence level in relation to technology, they ranged from limited skills to advanced user. The patients in the group that tracked activity with the fitness tracker explained that the device was easy to use. A participant mentioned the following:

You do not need any special technological skills to use it, it can be used by anyone, you only need to put it on your hand and off you go.

P7

The patient (P7) also described that he found the fitness tracker suitable for him because he felt that he had limited competence in mastering IT. He explained that it was important for him to have the right match between the required level of competencies and skills needed to operate the device and that the right match was the reason that he kept tracking.

In addition, some patients described an experience of autonomy related to having *the right* IT competencies. Some patients described that they had worked with technology and had thereby achieved IT competencies. The patients explained that when they experienced having the right level of IT competence, they found the device easier to use and were more likely to experiment with the devices. A patient said the following:

I am used to working with technology and find it exciting. So, I tried to place the cable differently and then it worked.

P6

It seemed that the experience of sustaining one’s self-image and autonomy occurred if the patients had prior and adequate competencies to use the mHealth devices and found the mHealth devices easy to use.

However, some patients did not feel competent to use these devices. During a coaching session, a patient (P3) expressed disinterest in using the device. He told the coach that he could not get the Bluetooth button to work and that he could not read the instructions because they were in English, so he returned the button to the coach. Likewise, a patient, in an interview, said that he became frustrated and insecure when he had to change the battery in the Bluetooth button, fearing that “it would explode” (P1). The sense of not having the proper competencies to use the mHealth devices seemed to frustrate the patients. It appeared that a feeling of being subject to paternalism could occur if the patients were forced to use the devices and did not possess the proper competencies to use them.

Some of the patients explained that they only used the devices to please the coach, and other patients found the mHealth device too abstract, particularly the music device. These patients explained that they would have liked to obtain more information about how to use the music device and the purpose of using it. In addition, a patient explained that he felt powerless because he could not see the point of using either the fitness tracker or the music device:

Maybe the fitness tracker supports being able to keep up with how much you walk, but I cannot see the point of having to play the music device in the evening. So, I did not use it.

P4

It seemed that patients experienced autonomy when they experienced possessing the required IT competencies. Consequently, when patients were unable to operate the devices meaningfully, they experienced reduced autonomy.

## Discussion

### Principal Findings

The objective of this study was to explore the experiences of patients with PCa and their perspectives on an intervention offering self-management support through mHealth and health coaching aimed at helping the patients achieve well-being. We applied the SDT by Ryan and Deci [18] as a theoretical framework to interpret and discuss our findings. Our analysis indicated that patients’ identity and self-image influenced their experiences with and perspectives on the self-management afforded by mHealth and health coaching in clinical practice. When the patients experienced having the suitable skills for the intervention, they were able to sustain their self-image, and then, they experienced well-being. This finding is in accordance with the SDT by Ryan and Deci [18,19]. Ryan and Deci [18] argued that the sense of self plays a significant role when new tasks and actions are required in new social contexts, and when self-awareness and esteem match the tasks at hand, people feel satisfied and well. Our findings are consistent with the identity-based motivation theory, which highlights that humans are motivated if they experience that their self-image fits with their current situation and circumstances and that humans prefer to act in ways that lead to sustaining their self-image rather than repressing it [20]. When the patients in this study experienced that the tasks and actions in the intervention fitted their identity, they felt that the tasks and actions became more meaningful to them, and their identity was sustained [18,19]. Moreover, when the tasks and actions did not align with the task at hand, the patients tried to steer toward not having to repress their identities. This caused some patients to reject using the devices or use their private devices [18]. According to Ryan and Deci [18], the fulfillment of autonomy, competence, and relatedness is essential for patients to achieve well-being. Our study indicates that each patient’s sense of self could be sustained if their basic psychological needs were fulfilled, which seemed to motivate the patients to participate in the intervention [18]. Our findings highlight that the patients seemed to be more motivated and satisfied when their actions were self-initiated; however, we also found that the patients were demotivated if they experienced being directed (rather than motivated) by the coach. Franklin et al [21] supported these findings and argued that to facilitate well-being in a self-management intervention, an autonomy-supportive environment is important to increase intrinsic motivation for the sustained self-regulation of health behavior. Furthermore, research has illuminated how autonomy is essential for achieving self-management goals and for improving health-related goals [22]. Our findings also highlight that the patients’ level of health literacy (eg, individual skills and technical competencies) should be taken into account when offering a self-management intervention through mHealth for the patients to successfully engage in self-tracking [18,23]. Patients face new tasks and require a new set of skills when using mHealth, which carries the additional challenge of digital health literacy [23]. As a consequence of the use of mHealth, the health care system risks excluding some patients [23]. This consequence was demonstrated in our study by some patients who declined to use the mHealth device when they could not understand the logic and rationale behind its use. In line with this, several studies have concluded that objective self-tracking data, such as activity or heart rate, are easy to capture and that patients understand and can convert the understanding of such data into actions that lead to healthy behavior [24]. This emphasizes the importance of the patients’ health literacy level in their understanding of the logic and rationale behind the tracking and, in accordance with this, making healthier choices.

Hilty et al [25] claimed that not all patients may be suitable for mHealth, and research shows that patients with lower health literacy are less likely to use different types of digital health tools than those with high health literacy. To include more patients in self-management interventions through mHealth and health coaching, the health care system must consider the patients’ different needs and skills when offering mHealth devices as a part of their care. Therefore, to facilitate a successful self-management intervention, it could be important to screen patients’ IT competencies before offering them an mHealth device, as Chan et al [26] suggested. Health care professionals are another pivotal element in the implementation of mHealth devices because they are important for the initiation and facilitation of self-management interventions during their interaction with patients [27]. In our study, we also found that the patients’ need for relatedness with the nurses (coaches) had implications on their well-being, and the patients expressed that it was important to have a feeling of belonging and being significant in the eyes of the nurses [18]. In this way, the role of the coach was important and motivating for the patients to translate data into everyday health-related advice and support for behavioral change. These findings are supported by a randomized trial that confirmed the importance of the interaction between patients and coaches as crucial in improving the well-being of patients [28]. This also correlates with a recent study showing that nurse-led coaching increases emotional well-being in patients with chronic diseases [29].

### Study Strengths and Limitations

As this study was designed within the framework of the ID methodology, as outlined by Thorne [16], it ensured that the research was both methodologically and interpretively rigorous. Methodological rigor and interpretive rigor are described as the 2 types of rigor required for qualitative research to be considered credible [30]. Epistemological integrity was achieved by ensuring that the design and implementation of this study were consistent with ID [16] and SDT. Credibility was enhanced through researcher triangulation. The author team consisted of people with various backgrounds in health care and research, which provided various perspectives on the analysis, interpretation, and understanding of the data.

Moreover, the credibility of our findings was enhanced through the triangulation of interview data and observational data, which strengthened analytical interpretations owing to the variety of insights [30]. We used participant observations and notes from the coaches to provide insights and challenge the interviews with the patients and to help interpret the patients’ experiences with and perspectives on the self-management intervention, which could have prevented placing an overemphasis on the individual interviews [31]. For example, the notes from the coaches provided unique insights regarding the interaction between the patients and the coaches and, in this way, added to the information gained through the interviews. The notes provided insight into the fact that the patients seemed more honest with the coach than with the interviewer. This may have meant that the opinions expressed by the patients during the interviews were more positive, but combined with the observational data, the interview data provided us with realistic insights into the patients’ perceptions of using mHealth. Overall credibility was sought through the use of consistent analytic logic [16]. The use of the same interview guide for all 26 interviews allowed the same structural and analytical consistency.

A limitation of the study is that it is unclear whether we examined the use of mHealth, health coaching, or both. Furthermore, a limitation could be that the health coaching was conducted in a room in the outpatient clinic, and the physical structures and social dynamics could have affected the interaction between the coach and the participant [32]. Research has pointed out that the environment in which coaching takes place is significant for facilitating a coaching conversation [32], and as our coaching was performed in the outpatient clinic, this could have resulted in a more traditional medical conversation, meaning that the potential of the coaching conversation was not fully used. Although purposive sampling does not confer transferability, it has provided in-depth information about patients with PCa in observational management and their experiences with and perspectives on the intervention [16]. On the basis of our description of the research context and our assumptions, we believe that our findings can be applied to patients with other cancer diseases in other health care settings in Western countries.

### Conclusions

For patients with PCa to achieve well-being, we found it important for them to be able to sustain their identity and self-awareness when offered a self-management mHealth intervention. The patients’ individual experiences and perspectives indicated that they placed themselves in a dynamic continuum of sustaining or repressing their identities and self-images. For the patients to sustain their self-image throughout the self-management intervention, motivation, autonomy, having the suitable competencies, and the interaction with the nurse coach appeared to be important aspects. In contrast, demotivation and paternalism could result in an experience of having to repress their identity and self-image, and thus, they have a negative effect on the use of mHealth. The patients seemed to be motivated to track when they felt in control, had the suitable competencies and skills related to technology, and when they experienced that their insights were significant to others.

To further understand the barriers and potential for developing a successful self-management intervention, future research should investigate health care professionals’ experiences with and perspectives on the use of mHealth and health coaching.

## Abbreviations

AS: active surveillance
ID: interpretive description
mHealth: mobile health
PCa: prostate cancer
SDT: self-determination theory
